# Using cognitive pre-testing methods in the development of a new evidenced-based pressure ulcer risk assessment instrument

**DOI:** 10.1186/s12874-016-0257-5

**Published:** 2016-11-16

**Authors:** S. Coleman, J. Nixon, J. Keen, D. Muir, L. Wilson, E. McGinnis, N. Stubbs, C. Dealey, E. A. Nelson

**Affiliations:** 1 Leeds Institute of Clinical Trials Research, University of Leeds, Leeds, UK; 2 Leeds Institute of Health Sciences, University of Leeds, Leeds, UK; 3 Mid Yorkshire Hospital NHS Trust, Wakefield, UK; 4 Leeds Teaching Hospitals NHS Trust, Leeds, UK; 5 Wound Prevention and Management Service, Leeds Community Healthcare NHS Trust, Leeds, UK; 6 School of Health & Population Sciences, University of Birmingham, Birmingham, UK; 7 School of Healthcare, University of Leeds, Leeds, UK

**Keywords:** Cognitive pre-testing methods, Risk assessment, Pressure ulcer, Tissue viability, Nursing, Patient and Public Involvement (PPI)

## Abstract

**Background:**

Variation in development methods of Pressure Ulcer Risk Assessment Instruments has led to inconsistent inclusion of risk factors and concerns about content validity. A new evidenced-based Risk Assessment Instrument, the Pressure Ulcer Risk Primary Or Secondary Evaluation Tool - PURPOSE-T was developed as part of a National Institute for Health Research (NIHR) funded Pressure Ulcer Research Programme (PURPOSE: RP-PG-0407-10056). This paper reports the pre-test phase to assess and improve PURPOSE-T acceptability, usability and confirm content validity.

**Methods:**

A descriptive study incorporating cognitive pre-testing methods and integration of service user views was undertaken over 3 cycles comprising PURPOSE-T training, a focus group and one-to-one think-aloud interviews. Clinical nurses from 2 acute and 2 community NHS Trusts, were grouped according to job role. Focus group participants used 3 vignettes to complete PURPOSE-T assessments and then participated in the focus group. Think-aloud participants were interviewed during their completion of PURPOSE-T.

After each pre-test cycle analysis was undertaken and adjustment/improvements made to PURPOSE-T in an iterative process. This incorporated the use of descriptive statistics for data completeness and decision rule compliance and directed content analysis for interview and focus group data. Data were collected April 2012-June 2012.

**Results:**

Thirty-four nurses participated in 3 pre-test cycles. Data from 3 focus groups, 12 think-aloud interviews incorporating 101 PURPOSE-T assessments led to changes to improve instrument content and design, flow and format, decision support and item-specific wording. Acceptability and usability were demonstrated by improved data completion and appropriate risk pathway allocation. The pre-test also confirmed content validity with clinical nurses.

**Conclusions:**

The pre-test was an important step in the development of the preliminary PURPOSE-T and the methods used may have wider instrument development application. PURPOSE-T proposes a new approach to pressure ulcer risk assessment, incorporating a screening stage, the inclusion of skin status to distinguish between those who require primary prevention and those who require secondary prevention/treatment and the use of colour to support pathway allocation and decision making. Further clinical evaluation is planned to assess the reliability and validity of PURPOSE-T and it’s impact on care processes and patient outcomes.

## Background

Pressure ulcers (PUs) are defined as ‘localised injury to the skin and/or underlying tissue usually over a bony prominence, as a result of pressure, or pressure in combination with shear’ [1]. They have a negative effect on patients quality of life [2, 3] and are costly to health care organisations world wide [4–7].

It is not appropriate to prevent PUs by subjecting all patients, including those not at risk to resource intensive interventions (such as repositioning by nurses, expensive mattresses), as these may impact on their quality of life (by disturbing sleep, for example) and divert nursing time from other essential areas [8]. Therefore we must target care appropriately, which is achieved by assessing the patient for the presence of PU risk factors (e.g. immobility, poor skin status, or poor perfusion), a process known as risk assessment. Risk assessment is considered the cornerstone to PU prevention and is recommended by international and national PU prevention guidelines [8–11]. To support clinical practice, Risk Assessment Instruments (RAIs) have been developed and are commonly used to systematically identify patients at risk, in preference to clinical ‘judgement’ of risk alone, despite the lack of evidence of their clinical effectiveness [1, 9, 10, 12, 13].

The theoretical basis of instrument development has grown over the last 30 years leading to the development and validation of a wide range of instruments designed to measure health status and quality of life [14]. Gold standard instrument development methods focus upon ensuring content validity and a conceptual framework, with testing and evaluation to establish psychometric properties including content, construct, convergent, discriminant, known group, criterion and concurrent validity, and inter-rater, intra-rater and test retest reliability [14–17]. In addition, as RAIs aim to predict those at risk of PU development it has been argued [18–20], much like other clinical prediction models (e.g. a predictive instrument to estimate the risk of mortality following cardiovascular surgery [21]), that their content needs to be informed by multivariable modelling with subsequent model testing on a ‘new’ prospective target population [22]. Establishing content validity is fundamental and needs to be a key consideration, as subsequent testing of other measurement properties will not replace or rectify problems with content validity [17]. Attention should also be given to the usability and acceptability of the instrument to users and patients. This is considered important in the development of instruments to facilitate content validity and ensure they are relevant, and understandable to the target population [14, 16, 17, 23–26].

Limitations of existing RAIs were identified following a systematic review of the risk factor literature [27] and a review of the content, development and testing of the 14 RAIs [16] included in the recent NICE review [10]. The instruments were developed for varying patient populations including acute hospital, intensive-care units, rehabilitation units and nursing homes. The review [16] identified that many instruments were developed decades ago when there was a paucity of PU risk factor evidence and a lack of methodological guidance for instrument development and evaluation [16]. Only two instruments reported a conceptual framework [28, 29] and the majority of instruments (*n* = 11) were developed on the basis of clinical opinion and/or literature review, or adaptations of original instruments [30–39]. The remaining 3 instruments were developed using statistical modelling methods, but are limited by the use of single centre populations, inadequate sample sizes and/or use of the same data set for development and validation [29, 40, 41]. These limitations have led to inconsistent inclusion of risk factors across instruments [16] and raise concern about their content validity. In addition, there is limited evidence that instrument developers involved the population of intended users, such as people at risk of pressure ulceration [16].

To address these limitations, a new evidence-based RAI, the PU Risk Primary Or Secondary Evaluation Tool - PURPOSE-T was developed as part of a National Institute for Health Research (NIHR) funded Programme Of Research (PURPOSE: RP-PG-0407-10056). The development of PURPOSE-T comprised 5 phases and incorporated innovative service user involvement:

1. Systematic review of epidemiological literature to identify risk factors associated with increased probability of PU development [27].
2. Consensus study incorporating an international expert group, evidence review and service user views to agree the risk factors, assessment items and structure of PURPOSE-T [42].
3. Proposal of a new PU conceptual framework to show the critical determinants of PU development and theoretical causal pathway and to underpin PURPOSE-T [43].
4. Design and pre-testing of the draft PURPOSE-T to confirm content validity assess and improve the acceptability and usability [8]﻿.
5. Clinical evaluation of 230 patients from acute and community settings to assess reliability, validity (convergent and known groups), data completeness and clinical usability [8].

The first 3 phases of this work and the support of a graphic designer underpinned the development of the initial draft of PURPOSE-T, comprising a 3-step risk assessment process. In Step 1, a screening stage, the patient’s mobility and skin status are assessed with yes/no items and one of two decisions are made: not at risk OR potentially or definitely at risk - hence continue to Step 2. In Step 2, a full assessment stage, 7 risk factors of Immobility, PU and Skin Status, Perfusion, Diabetes, Moisture, Sensory perception and Nutrition are assessed using yes/no items. The items in Step 1 and 2 are colour coded and based upon the responses and colour coding for each item the assessor is required, in Step 3, to identify one of three decision pathways: secondary prevention and treatment pathway (for those with an existing PU or with scarring from a previous PU); primary prevention pathway (for those at risk of a PU) OR; not currently at risk pathway [16]. The final version of PURPOSE-T can be accessed via: http://medhealth.leeds.ac.uk/accesspurposet.

This paper describes the phase 4 pre-test and highlights the impact of including a pre-test stage as part of the instrument development process.

### Aims

The aim was to assess and improve the acceptability, usability, format, design, clarity, comprehension, language and data completeness of the draft PUPROSE T with clinical nurses [8]. While content validity was a key consideration of the consensus study, the pre-test also aimed to confirm the content validity with intended end users, clinical nurses.

## Methods

The pre-test involved cognitive pre-testing methods to evaluate how clinical nurses interpreted questions, response categories and instructions while using the draft PURPOSE-T [8, 44], and innovative integration of service user views via the PU Research Service User Network (PURSUN UK: http://www.pursun.org.uk/) including co- development of vignettes to facilitate pre-testing and the review of PURPOSE-T following pre-testing. Cognitive pre-testing methods are well established in the development of health status and patient reported outcome measures and are considered important for improving precision, confirming content validity and ensuring the instrument is understood and relevant to the target population [14, 17, 25, 26].

The specific pre-testing methods used in this study incorporated focus groups and one-to-one think-aloud interviews. A focus group is a group interview which incorporates group interaction as part of the method and is useful in exploring peoples, knowledge, attitudes and experience [45]. One-to-one think-aloud interviews encourage participants to vocalise their thoughts or ‘think-aloud’ while they are concurrently undertaking a task [46], in this case completion of the PURPOSE-T. The use of both focus groups and one-to-one think-aloud interviews was considered appropriate, as it was anticipated that the focus groups would allow more general usability issues to be identified e.g. relating to the implementation of PURPOSE-T, while the think-aloud interviews would allow more specific item issues to be identified e.g. difficulties in understanding the wording of items within PURPOSE-T [47, 48]. Both would aid instrument development and eventual implementation.

**Fig. 1:**
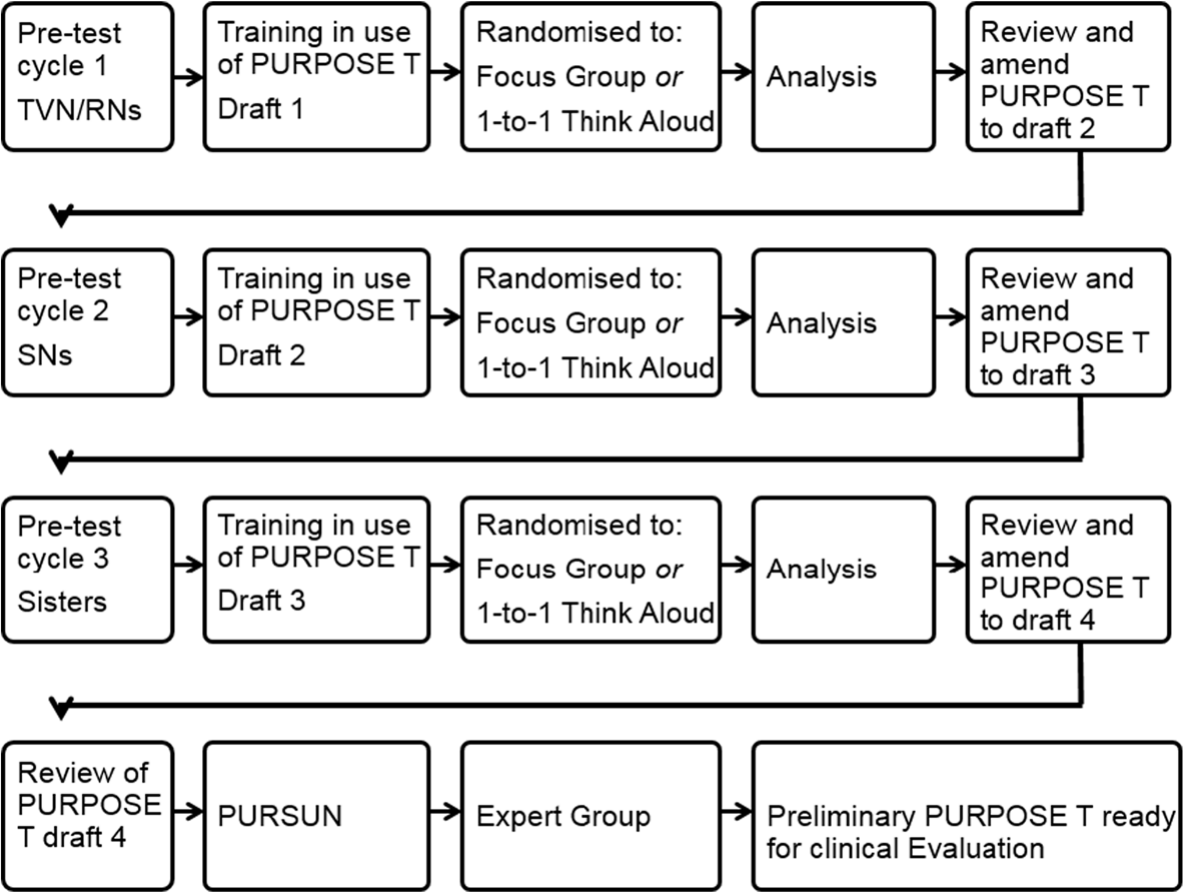
Pre-test Cycles

The pre-test study was conducted over three cycles each comprising participant training in the use of the draft PURPOSE-T, followed by a focus group and one-to-one think-aloud interviews (Fig. 1). Purposive sampling was undertaken to ensure that Tissue Viability Nurses (TVNs), Staff Nurses (SN) and Sisters/charge nurses) with an interest in tissue viability (e.g. a link nurse or member of a local PU or wound care working group), from 2 acute (1 district general hospital and 1 large teaching trust) and 2 community NHS Trusts in West Yorkshire UK, were grouped according to job role (cycle 1: TVNs; cycle 2: SN; cycle 3: Sister/Charge Nurses) [8]. It was anticipated that this would facilitate openness, as heterogeneous groups can lead to inhibition in raising issues that do not seem to be shared by others [8, 49, 50]. This was thought to be particularly important for this group as a hierarchy might have stifled disclosure (e.g. a staff nurse might not want to disagree with the views of their Sister/Charge Nurse) [8]. Having nurses from different centres minimised familiarity which can lead to participants relying on ‘taken for granted’ assumptions [8, 50].

In each pre-test cycle 3 focus groups comprising four to eight nurses [45] was undertaken to allow important general themes [51] associated with the implementation of PURPOSE-T to be identified. In addition, we estimated that approximately 4 think- aloud interviews for each of the three pre-test cycles (each interview incorporating the completion of 3 PURPOSE-T assessments using different vignettes) were needed to reach saturation, with no new issues arising. This was slightly lower than cognitive interviewing guidance for questionnaire development indicating a suitable sample size of 5–15 individuals per interviewing round [48], but this sample size was thought to be sufficient in light of the low number of data items in the PURPOSE-T and the concurrent focus group data collection.

After each pre-test cycle, analysis was undertaken and the results reviewed by a clinical/academic Working Group (authors) who agreed adjustment/improvements to the draft PURPOSE-T. These were implemented in the next cycle in an iterative process (Fig. 1). Following the final pre-test cycle, PURPOSE-T was reviewed by PURSUN UK members who comprise patients and carers with direct experience of living with PUs or PU risk. PURSUN was set up to improve the quality of Patient and Public Involvement (PPI) in PU research. PURPOSE-T was also reviewed at a meeting of the international expert group (comprising clinical/academic experts in the PU field including nurses, doctors, bioengineers, an epidemiologist, and individuals with organisational development and clinical decision making expertise) involved in the earlier instrument development consensus study phase [42].

### Data collection

Data collection was undertaken between April 2012 and June 2012. The pre-tests were held away from the clinical area in a comfortable university setting, lasted 2.5 h in total with refreshments available throughout [45]. The initial training, undertaken by all participants involved a short presentation and demonstration of how to use the draft PURPOSE-T with a simulated patient [8]. Each nurse then completed the draft PURPOSE-T using simulations of real patient situations via written vignettes. The vignettes were case studies comprising key patient characteristics pertinent to PU risk assessment and were designed to allow participants to practice using PURPOSE-T and to prompt study participant responses [52] while undertaking think-aloud interviews or focus groups [8]. These were accompanied by photographs of PUs and were appropriate to the nurses’ area of practice (i.e. community nurses used vignettes of community patients) [8]. The vignettes comprised 7 acute and 7 community case studies and were co-developed by the researcher (SC), the Working Group and members of PURSUN prior to the pre-test to ensure they were realistic and clinically relevant. They incorporated a range of patients including those not currently at risk, those at risk and those with existing PUs to maximise the potential of identifying usability issues with PURPOSE-T over the course of the pre-test [8].

Nurse participants were then assigned to either a focus group or a one-to-one think-aloud interview which were audio-recorded to allow analysis to facilitate the development PURPOSE-T. The assignment to a focus group or a one-to-one think-aloud interview was undertaken randomly to make it fair to participants, as it was anticipated that the focus groups would be favoured by nurses. Those allocated to the focus group were asked to independently complete PURPOSE-T again, using 3 of the 7vignette case studies relevant to their area of practice prior to the focus group meeting. The specific vignettes were assigned to each nurse to ensure all 7 were used by at least 1 participant at each focus group. Nurse participants were encouraged to highlight any areas they found confusing on the PURPOSE-T form. A co-facilitator assessed data completeness and listed areas where data items were not completed or not completed as required, as well as areas noted by the nurses as confusing [8]. The focus group meeting then convened to discuss the use of PURPOSE-T. The facilitator (SC) promoted group interaction and guided discussions around a topic guide, which considered the usability and areas of confusion regarding the use of PURPOSE-T, as well as any anticipated problems with using it in clinical practice [8]. This was informed by the data completeness assessment.

Up to four different nurses from each pre-test cycle were randomly assigned to the one-to-one think-aloud interview. A researcher (EMc or DM) conducted the interviews around a topic guide. Firstly, the nurse participants were guided through the think-aloud technique. Once the nurses were content with the approach, they were asked to complete PURPOSE-T again using 3 of the 7 vignette case studies appropriate to their area of practice in the presence of the researcher. The specific vignettes were assigned to each nurse to ensure all 7 were used by at least 1 think-aloud participant at each pre-test cycle. The researcher encouraged the nurses to vocalise their thoughts as they completed the PURPOSE-T and were able to probe less vocal participants concurrently or retrospectively regarding completions of the task [8, 46, 48]. Potential scripted probes were prepared in advance, but interviewers were also at liberty to use spontaneous probes as relevant to the particular interview.

### Ethical considerations

The study was reviewed for potential ethical hazards and approved by a University of Leeds Ethics Committee. Informed consent was obtained prior to study participation and participants were free to withdraw from the study without giving reasons.

### Data analysis

To assess data completeness and compliance with decision rules, PURPOSE-T assessments were analysed using descriptive statistics after each pre-test cycle for assessments where only Step 1 (Screening) were completed and assessments where both Step 1 (Screening) and Step 2 (Full Assessment) were completed. Summaries included: number and percent of item level missing data; number and percent of risk categories allocated and; number and percent items missing where a risk category had been allocated.

Focus group meetings and the think-aloud interviews were transcribed verbatim. The researcher (SC) listened to the audio-tapes and read the transcripts to ensure accuracy and that they had a good overview of the focus group discussions and one-to-one think-aloud interviews [8]. The data was then manually coded by the researcher (SC) with initial categories based on the items of the draft PURPOSE-T, using a directed content-analysis approach [53] with additional codes being added as they emerged from the data. This was reflected in summary reports, which were reviewed by the focus group facilitators and the think-aloud researchers to ensure it reflected discussions/interviews [8]. The emphasis was on identifying themes about content, format, design, comprehension and language across the focus groups and think-aloud interviews which impacted on the acceptability, data completeness and usability of PURPOSE-T in clinical practice.

## Results

Over the three pre-test cycles 34 nurses from acute (*n* = 16) and community settings (*n* = 18) participated. Table 1 details the characteristics of the nurses involved.

**Table 1:** Demographic details of pre-test participants

	Pre-Test Session 1 (TVN/RNs)	Pre-Test Session 2 (Staff Nurse)	Pre-Test Session 3 (Sisters)	Overall/total
	N:11	N: 12	N: 11	N: 34
Age (years)	Range: 30–55 Median: 41	Range:23–59 Median: 37	Range 34–54 Median: 50	Range: 23–59 Median: 46.5
Gender	Female: 10 Male: 1	Female: 12 Male: 0	Female: 11 Male: 0	Female: 33 Male: 1
Nationality	British: 10 Malaysian: 1	British: 12	British: 10 Polish: 1	British: 32 Malaysian: 1 Polish: 1
NHS Acute Sector	DGH 3 THT: 3	DGH: 2 THT: 3	DGH: 3 THT: 2	DGH: 8 THT: 8
NHS Community Sector	CT 1: 4 CT 2: 1	CT 1: 4 CT 2: 3	CT 1: 3 CT 2: 3	CT 1: 11 CT 2: 7
Roles	TVN:7 TV RN: 4	SN:11 SM:1	Acute Sister: 5 DN Sister: 2 DN CL: 2 DN Practice educator: 2	

*N* Number, *DGH* District General Hospital Trust, *THT* Teaching Hospitals Trust, *CT* Community Trust, *DN* District Nurse, *CL* Clinical Lead, *SN* Staff Nurse, *SM* Staff Midwife

At each pre-test cycle, four nurses undertook think-aloud interviews (12 nurses over the 3 pre-test cycles) and seven or eight nurses attended the focus groups (22 nurses over the 3 pre-test cycles) [8]. They completed 101 PURPOSE-T assessments using vignette case studies; in the first pre-test 11 TVN/Research Nurses undertook 32 PURPOSE-T assessments, in the second pre-test 12 staff nurses undertook 36 PURPOSE-T assessments and in the third pre-test 11 Sisters undertook 33 PURPOSE-T assessments [8].

**Fig. 2:**
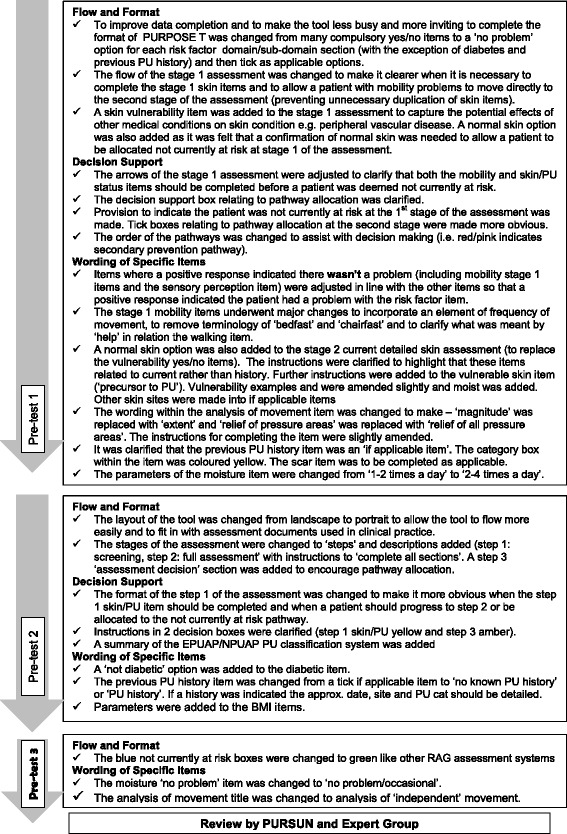
Changes to PURPOSE-T following each pre-test sessions [8]

Changes made to the PURPOSE-T between pre-test cycles are summarised in Fig. 2 and include three main areas:

- flow and format, i.e. colour coding, layout and order of assessment items.
- decision support, i.e. instructions to facilitate appropriate assessment and pathway allocation.
- wording of specific items.

**Fig. 3:**
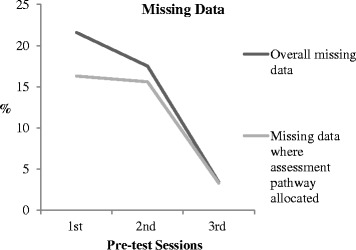
Percentage of missing data at each pre-test session [8]

**Fig. 4:**
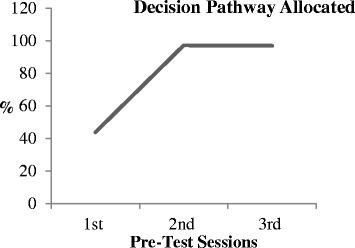
Percentage decision pathway allocated at each pre-test session [8]

The majority of changes were made following pre-test cycle 1, with ongoing refinement following pre-test cycle 2 and minimal adjustments following pre-test cycle 3 (Fig. 2). The qualitative and quantitative data were interpreted simultaneously in order to understand the impact of the format, flow, decision support and wording upon data completeness to inform amendments to the draft instrument [8]. For example, the data completeness assessment in the first pre-test (Tables 2 and 3 and Figs. 3 and 4) showed high levels of missing data and poor levels of decision pathway allocation [8]. The corresponding focus group discussions highlighted confusion about where to indicate pathway allocation: I think putting the tables here seperates the key discussion points.

**Table 2:** Item level completion for assessments that included step 1 (screening) and step 1 (screening) and 2 (full assessment) [8]

	Pre-test 1: N items requiring completion p/a	Pre-test 1: (TVN/RNs) Items completed	Pre-test 2: N items requiring completion p/a	Pre-test 2: (Staff Nurse) Items completed	Pre-test 3: N items requiring completion p/a	Pre-test 3: (Sisters) Items completed
Item level completion for assessments that included step 1 (screening)						
Mobility	4	100% (24/24)	At least 1 of 4	100% (10/10)	At least 1 of 4	100% (8/8)
Skin/PU status	2	66.7% (8/12)	At least 1 of 4	90% (9/10)	At least 1 of 4	100% (8/8)
Decision pathway allocated	1	0% (0/6)	1	100% (10/10)	1	87.5% (7/8)
Total Item completion	-	76.2% (32/42)	-	96.7% (29/30)	-	95.8% (23/24)
Total Item completion where decision pathway allocated	-	0%	-	96.7% (29/30)	-	100% (21/21)
Item level completion for assessments that included step 1 (screening) and 2 (full assessment)						
Mobility (1^st^ stage)	4	93.3% (97/104)	At least 1 of 4	96.2% (25/26)	At least 1 of 4	100% (25/25)
Skin/ PU status (1^st^ stage)	2	98.1% (51/52)	AA	100% (3/3)	AA	100% (1/1)
Movement Matrix	1	100% (26/26)	1	100% (26/26)	1	96% (24/25)
Sensory Perception	1	96.2% (25/26)	1 of 2	100% (26/26)	1 of 2	100% (25/25)
Current DSA - listed sites	15	71.5% (279/390)	13	75.4% (255/338)	13	97.2% (316/325)
Current DSA – other sites	AA	0% (0/0)	AA	50.0% (1/2)	AA	0% (0/0)
Current PU	AA	84.2% (16/19)	AA	83.3% (20/24)	AA	80.0% (20/25)
Previous PU history	AA	75% (9/12)	AA	77.8% (7/9)	1 of 2 (if yes 3, AA)	85.3% (29/34)
Scarring	2	55.8% (29/52)	AA	100% (1/1)	AA	100% (1/1)
Perfusion	2	92.3% (48/52)	At least 1 of 3	73.1% (19/26)	At least 1 of 3	100% (25/25)
Nutrition	4	76.9% (80/104)	At least 1 of 5	100% (26/26)	At least 1 of 5	100% (25/25)
Moisture	1 (if yes 2 as applicable)	74.1% (40/54)	1 of 3	84.6% (22/26)	1 of 3	100% (25/25)
Diabetes	1	100% (26/26)	As applicable	100% (5/5)	1 of 2	100% (25/25)
Decision pathway allocated	1 of 3	53.8% (14/26)	1 of 3	96.2% (25/26)	1 of 3	100% (25/25)
Total Item completion	-	78.5% (740/943)	-	81.7% (461/564)	-	96.6% (566/586)
Total Item completion where decision pathway allocated	-	83.7% (417/498)	-	83.7% (452/540)	-	96.6% (566/586)

*N* number, *p/a* per assessment, *AA* as applicable, *DSA* detailed skin assessment, *TVN* tissue viability nurse, *RN* research nurse, *PU* Pressure Ulcer

**Table 3:** Overall total Item completion for assessments

	Pre-test 1: (TVN/RNs) Items completed	Pre-test 2: (Staff Nurse) Items completed	Pre-test 3: (Sisters) Items completed
Overall total Item completion for assessments			
Total item completion for assessments concluding at step1	76.2% (32/42)	96.7% (29/30)	95.8% (23/24)
Total item completion for assessments including step 1 and 2	78.5% (740/943)	81.7% (461/564)	96.6% (566/586)
Overall total item completion	78.4% (772/985)	82.5% (490/594)	96.6% (589/610)
Overall total Item completion for assessments with decision pathway allocated			
Total item completion for assessments concluding at step1 where decision pathway allocated	0%	96.7% (29/30)	100% (21/21)
Total item completion for assessments including step 1 and 2 where decision pathway allocated	83.7% (417/498)	83.7% (452/540)	96.6% (566/586)
Overall total Item completion where decision pathway allocated	83.7% (417/498)	84.4% (481/570)	96.7% (587/607)

TVN tissue viability nurse; RN research nurse

Focus group participant - ‘yes I got to that point [the assessment decision section] and then finished it and ignored those boxes at the bottom really’.

Some nurses had attempted to indicate a pathway though they were clearly unsure of where to do this. This brought to light a significant omission and lack of clarity within the PURPOSE-T, and the need to include a response box within the ‘not currently at risk’ pathway at step 1 of the assessment, and to make the pathway allocation response box at step 3 of the assessment more obvious [8].

The think-aloud interviews in the first pre-test session highlighted a complementary issue relating to the decision support and the ordering of the decision pathway boxes in the first draft PURPOSE-T. The original order was a blue ‘not currently at risk pathway’ on the left, an amber ‘at risk’ primary prevention pathway in the middle and the red ‘has a pressure ulcer’ secondary prevention/treatment pathway on the right. Confusion regarding this was highlighted by one participant as below:

Think aloud participant - ‘there is a box of where a patient is at risk, you know … the orange box where I’ve ticked … and because it comes first then I would have automatically ticked it and not bothered to look at the pink box. It’s not very clear … well, it’s not clear at all. So, I mean … probably, I don't know, swap it round so that … or, you know, from top to bottom, so you look through the higher risk, the red bit is first, then if red is not ticked, people will go down to the orange one…’.

The boxes were subsequently re-ordered so that the red was left (and therefore first), amber middle and blue right [8]. The blue box was changed to green following a subsequent pre-test cycle in line with other clinical assessments which incorporate red, amber, green (RAG) formats.

There were other examples of the two approaches (focus groups and think aloud interviews) providing complementary insights. When considering item wording the focus groups highlighted general issues relating to acceptability of terminology while the think-aloud participants provided more detailed information about areas of confusion. This is shown in the examples below:

1. Focus group participant 1 – ‘The terminology’s [primary and secondary prevention] not frequently used, is it?’ Focus group participant 2 – ‘No, I mean it’s something I’m quite familiar with, because I come from a background of stroke and TIA, so we used to use primary prevention, you know, for patients who had not had an event yet but were at risk, and then secondary prevention for a patient who had had an event and then. But I don’t know that it’s something that we’d automatically think of in this [PU management].

2) Think aloud participant - ‘So stage 2, looking at sensory perception. Does the patient feel and respond appropriately from pressure? There’s no indication of any sort of neurological problems, so I would say yes, she responds appropriately. The only thing I can think of is I have to think twice, because it’s what I’d describe as a double negative, it’s…. does the patient feel and respond appropriately to discomfort…… I mean, the sort of normal reaction is, the patient is fine so it’s not a problem, so in my opinion just a mistake to put a no. But…..so reading it, it’s actually yes, they respond appropriately to discomfort’ Interviewer - so you’re perhaps used to when there’s a yes answer, there’s a problem? Think aloud participant - yeah, yeah. So then yeah, you have to spend a bit of time to just think about it, so it may be a problem when you’re doing it quickly, I think’

The wording of the sensory perception item was changed prior to cycle 2 [8].

A more general issue highlighted by the participants of the first pre-test focus group was that they felt there should be some provision within step 1 of the PURPOSE-T to enable nurses to use their clinical judgement as detailed below:

Focus group participant - ‘We’ve always said about, you know, these are just tools, that you should use nursing judgement as well, is there any way you could have an extra box that says, you know, to the nurses, in your judgement, is this patient at risk?’ This may apply to the severity of a risk factor (e.g. severe diabetes, perfusion problems and severe nutritional problems) or other significant factors which may be exceptions to the rule. Having ‘other items’ at step 1 was considered by the Working Group but there was concern that the screening stage could become too large [8]. Taking into account the causal pathway for PU development [54], it was decided that it wasn’t the presence of these factors per se that was important, rather it was the impact they may (or may not) have on skin status and a ‘vulnerable skin’ item was therefore added to the skin status section of the screening stage.

The focus groups also provided more insight into potential future implementation issues that may arise e.g. one participant articulated concern regarding the use of colour in PURPOSE-T:

Focus group participant - ‘we’ve got a mega problem in community, because our documentation has to be photocopied and we don’t have a colour photocopier’.

Another participant highlighted the need for simplicity to facilitate implementation:

Focus group participant - ‘it’s got to be something that’s easier to implement, because we’re not going to be able to train every single nurse, so you want something … although you can try and train everyone, a lot of them aren’t going to have time to be relieved from the ward for training, so it’s something that they should be able to just pick up and start doing easily …’

The quantitative data analysis was useful in assessing the impact of the changes to data completion and pathway allocation over the three pre-test cycles (Tables 2, 3and 4) [8]. Figure 3 illustrates how the levels of missing data decreased over the 3 pre-test cycles overall and where a decision pathway was allocated. Figure 4 illustrates how the number of decision pathways allocated increased from the first to the second pre-test cycle. Table 4 presents the appropriateness of the decision pathways allocated according to the item responses for each assessment and compliance with the decision rules of PURPOSE-T.

**Table 4:** Appropriate decision pathway allocation [8]

	Pre-Test Session 1 (TVN/RNs)	Pre-Test Session 2 (Staff Nurse)	Pre-Test Session 3 (Sisters)
Appropriate pathway allocation	78.6% (11/14)	91.4% (32/35)	90.6% (29/32)
Inappropriate pathway allocation	7.1% (1/14)		
Pathway allocated but some uncertainty of appropriateness due to missing data items	14.3% (2/14)	8.6% (3/35)	9.4% (3/32)

*TVN* Tissue Viability Nurse, *RN* research nurse

### Review by PURSUN and Expert Group

The review of the PURPOSE-T by PURSUN UK and the expert group following the third pre-test cycle led to a final change to the PURPOSE-T. While PURSUN felt that the PURPOSE-T was clear and understandable, they raised concern about the wording of the sensory perception item relating to the ‘ability to feel and respond’ aspect of the item [8]. The group agreed that the patient would be at risk of PU development if they fulfilled one component of ‘ability to feel and respond’, but the wording suggested that it would only be a risk if both applied to the patient. They felt that the terminology should be ‘feel and/or respond’ [8]. This led to the rewording of the sensory perception item at the subsequent expert group meeting.

## Discussion

By using cognitive pre-testing methods we were able to assess and improve the usability and confirm the content validity of a new evidence-based RAI with clinical nurses over the course of three cycles. Pre-testing was particularly important given the increased support for decision making and instructions that are integrated in the PURPOSE-T. Nurse insights captured in the focus group discussions/think aloud interviews and measurement of instrument item level completion and appropriate pathway allocation facilitated the identification of areas of confusion. The combined methods ensured that all aspects of usability were considered and led to key changes to PURPOSE-T in three main areas including the flow and format, decision support and the wording of specific items. The pre-test was an important methodological step to instrument development and led to the preliminary PURPOSE-T in readiness for onward clinical evaluation of reliability and validity. This is the first study that we have been able to identify which fully reports pre-testing as a key methodological component in the development of a PU RAI and is underpinned by methodologies used in the development of other health measurement Instruments and patient reported outcome measures [14, 17, 25, 26, 55]. The involvement of clinical nurses has only been reported briefly in the literature for other RAI development [33–36] and evidence of service user involvement is lacking [16]. Previous developed instruments used methods which did not incorporate structured pre-testing methods as used in the development of PURPOSE-T and reported in this study. These could be important omissions as we found using a structured approach to assess and improve the usability and confirm the content validity [17] of PURPOSE-T with clinical nurses informative, potentially impacting on its eventual implementation in clinical practice.

The approach was supported by the involvement of PURSUN in developing realistic vignettes and reviewing PURPOSE-T following pre-testing, which led to important changes to shape the instrument. The acceptability of PURPOSE-T to patients and carers is important as risk assessment should involve consultation with the patient to facilitate shared decision making about risk and the use appropriate preventative interventions [56].

To support the aims of the pre-test, purposive sampling was used to target nurses from acute hospital and community settings, with a range of job roles (i.e. Tissue Viability Nurses, Staff Nurses and Sisters/Charge Nurses) and an interest in tissue viability. The age range and gender of nurse participants largely reflects national workforce trends [57], though in keeping with other research ethnic minorities were under represented [58–61]. It is unclear whether this impacted the usability issues raised in the pre-test, though the ongoing evaluation of PURPOSE-T will involve testing with a larger number of nurses in clinical practice and give further opportunity for instrument refinement.

Nurses being grouped in similar roles, prevented hierarchical issues impeding group member involvement in the cycles and was felt to facilitate greater disclosure [49, 50]. The order of the pre-test cycles (in terms of nurse job roles) was carefully considered to ensure usability issues were identified as quickly as possible, so that changes could be made to the draft PURPOSE-T and pre-tested in the subsequent cycle. The TVNs pre-test cycle was conducted first as it was anticipated that as specialist nurses in the PU field, they were best placed to identify any subject specific and key usability issues which could be addressed in subsequent versions. Additionally, the third and last pre-test cycle deliberately involved Sister/charge nurses so that PURPOSE-T (incorporating changes that were made in response to pre-test 1 and 2) could be considered by senior nurses responsible for patient care. The chosen order (of the nurses job role) was associated with a the decreasing number of changes made to the PURPOSE-T (Fig. 2) which is also indicative that saturation had been reached. However in the absence of comparisons with other role-orders we cannot determine if this could be attributed to this particular order.

The use of both focus groups and think-aloud interviews is unusual for evaluation purposes, but this is mainly due to differences in backgrounds and cultures of researchers which use the techniques (Willis 2005). The use of both techniques in the context of developing the PURPOSE-T was advantageous as while there was some overlap between the groups in terms of the nature of the issues raised (i.e. both groups identified issues relating to specific usability and wider application), they provided complementary insights. As previously noted by others the think-aloud interviews most consistently highlighted specific usability issues (e.g. relating to the wording of specific items) while the focus groups most consistently identified general level usability issues relating to the wider application/implementation of PURPOSE-T in clinical practice [47, 48]. In addition, the completion of 3 PURPOSE-T assessments by each participant and descriptive analysis of data completeness, highlighted problems with particular item completion and was useful in assessing the impact of the changes between versions. The methods used in this pre-test may have wider application, in the development and evaluation of other health related instruments.

PURPOSE-T proposes a new approach to PU risk assessment based on up to date evidence and robust development methods. Key features of PURPOSE-T include a screening stage to quickly screen out those not at risk, the inclusion of skin status to allow the distinction between those at risk who require primary prevention and those with an existing ulcer (or scarring from a previous ulcer) who require secondary prevention/treatment, and the use of colour, rather than traditional numerical scores to support pathway allocation and decision making. Future studies are required to evaluate whether the use of PURPOSE-T encourages individualised care planning in response to the patients specific risk profile and its impact on care processes and outcomes.

### Limitations

It could be argued that undertaking a pre-test using vignette case studies, is no substitute for assessing the PURPOSE-T in clinical practice. A limitation of this approach, with vignettes, is that is an artificial situation and it is acknowledged that participants may have responded differently in a real life situation [62]. However, the need to assess and improve the acceptability of the PURPOSE-T with clinical nurses was considered a robust and logical step to ensure content validity and usability, prior to evaluation in clinical practice with patients. In addition, the vignettes were co-developed by the project lead, the Working Group and members of PURSUN to ensure they were realistic, clinically relevant and to give an indication of external validity [63]. The use of vignettes has been used previously by social scientists in various fields [63], in dental, medical and nursing education [64–66] and to establish the validity of RAIs [67, 68]. In keeping with those who have used vignettes previously, the present study, benefitted from the approach, allowing exploration of participants knowledge, attitudes and how they might respond to a simulated event [62, 69].

In this study the transcription data from the focus groups and one-to-one think aloud interviews were manually coded and it might be argued that qualitative data software would have been a more robust means of managing transcription data. This relates to the assertion that the software packages provide a more transparent, auditable approach to coding which provides the basis for establishing credibility, though there is concern that use of such software encourages a focus on quantity and breadth rather than depth and this can distance the researcher from the data [70]. The decisions regarding qualitative software utilisation are influenced by the size and scope of the project and the expertise of the researcher [70, 71]. As the scope and size of this study was quite focussed, the use of manual data coding with summary reports being checked by other researchers involved in the process was considered an appropriate approach.

## Conclusion

This is the first study we are aware of that incorporates pre-testing with clinical nurses and the involvement of service users in the development of a RAI. The pre-test was an important development stage of this new instrument as it allowed important usability issues to be identified and addressed, and content validity to be confirmed with its intended end users. This was facilitated by the use of realistic vignettes that were co-developed with service users. While there was some overlap between the discussions of the focus groups and one-to-one think-aloud interviews, overall they were found to be complementary. The methods described in this pre-test may have wider health related instrument development application.

The pre-test led to the development of a preliminary PURPOSE-T in readiness for subsequent clinical evaluation to assess the validity and reliability of the RAI in practice. PURPOSE-T proposes a new approach to PU risk assessment, incorporating a screening stage to quickly screen out those not at risk and the use of colour to support pathway allocation and decision making. PURPOSE-T also incorporates skin status to facilitate a distinction between those who require primary prevention and those who require secondary prevention/treatment. Further study is needed to assess whether anticipated benefits of PURPOSE-T, including individualised care planning will lead to improved care processes and outcomes.

## Abbreviations

PU: Pressure ulcer
PURPOSE-T: Pressure ulcer risk primary or secondary evaluation tool
PURSUN: Pressure Ulcer Research Service User Network
PPI: Patient and public involvement
RAI: Risk assessment instrument
TVN: Tissue viability nurse
